# Supplementary material for: Recurrent exon-deleting activating mutations in AHR act as drivers of urinary tract cancer
Source: Sci Rep. 2022 Jun 16;12:10081. doi: 10.1038/s41598-022-14256-0 (PMC9203531; doi:10.1038/s41598-022-14256-0)
Supplement: Supplementary file 1 — Supplementary Information 1. [file 41598_2022_14256_MOESM1_ESM.pdf]

Supplementary information to: Recurrent exon-deleting activating mutations in *AHR* act as drivers of urinary tract cancer

Judith M. Vlaar[1], Anouska Borgman[2], Eric Kalkhoven[2], Denise Westland[1], Nicolle Besselink[1], Charles Shale[3,4], Bishoy M. Faltas[5], Peter Priestley[3,4], Ewart Kuijk[1,6,7], Edwin Cuppen[1,3,7,8]

[1] Center for Molecular Medicine and Oncode Institute, University Medical Center Utrecht, the Netherlands.

[2] Center for Molecular Medicine, University Medical Center Utrecht, the Netherlands.

[3] Hartwig Medical Foundation, Amsterdam, The Netherlands.

[4] Hartwig Medical Foundation Australia, Sydney, New South Wales, Australia.

[5] Department of Medicine and Department of Cell and Developmental Biology, Weill Cornell Medicine, New York, NY, United States.

[6] Division of Pediatric Gastroenterology, Wilhelmina Children's Hospital, University Medical Center Utrecht, the Netherlands

[7] These authors contributed equally to this work: Ewart Kuijk, Edwin Cuppen

[8] contact: e.cuppen@hartwigmedicalfoundation.nl

Competing interests

The authors declare no competing interests.

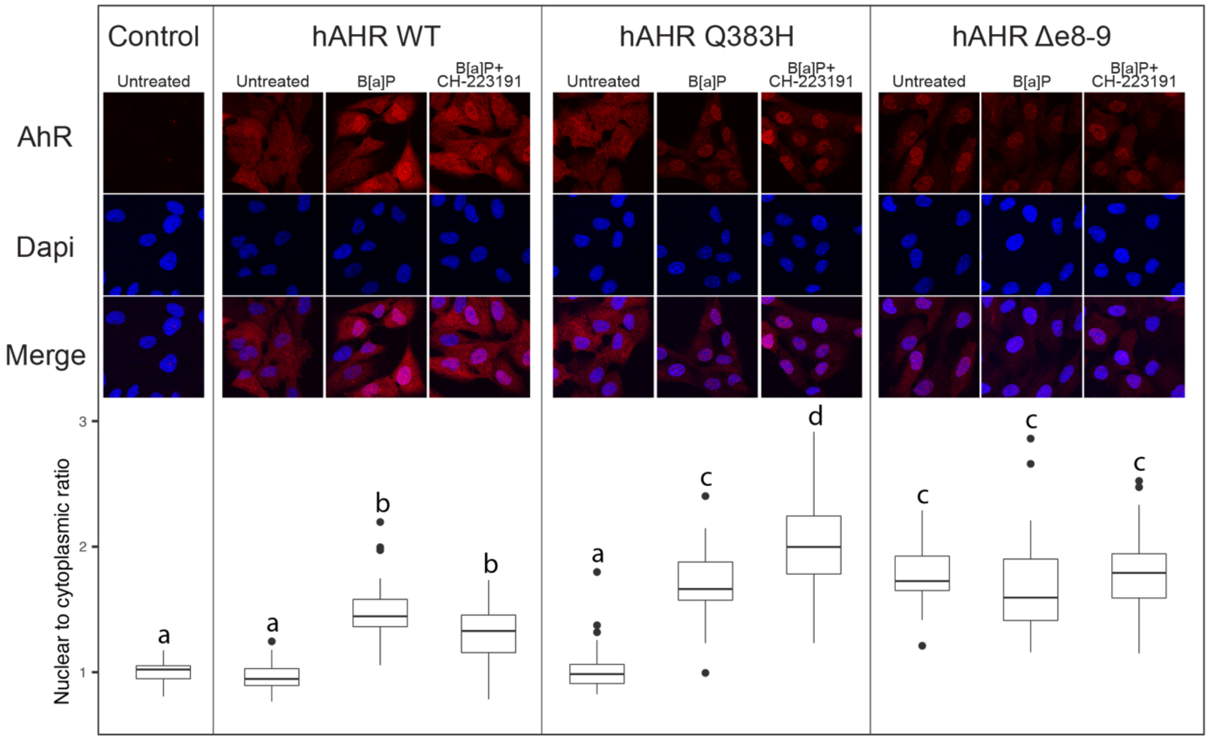

Supplementary figure 1: AHR protein localization after incubation with B[a]P or B[a]P and CH-223191.

Immunofluorescence of RPE1 cells that express *hAHR*^{WT}, *hAHR*^{Q383H}, or *hAHR*$^{\Delta e8\text{-}9}$ that were incubated for 4h with B[a]P or with AHR antagonist CH-223191. AHR was detected with immunofluorescence (red) and DNA with Dapi (blue). The boxplots present the AHR signal ratio of nuclear to cytoplasmic signal for all individual captured cells. Untransduced cells served as negative controls (left panels).
